# Two-stage hybrid Ivor-Lewis esophagectomy as surgical strategy to reduce postoperative morbidity for high-risk patients

**DOI:** 10.1007/s00464-020-07485-9

**Published:** 2020-03-12

**Authors:** I. Bartella, S. Brinkmann, H. Fuchs, J. Leers, H. A. Schlößer, C. J. Bruns, W. Schröder

**Affiliations:** Department of General, Visceral and Cancer Surgery, University of Cologne, Kerpener Str. 32, 50937 Cologne, Germany

**Keywords:** Esophageal cancer, Esophagectomy, Ischemic conditioning, Postoperative outcome

## Abstract

**Background:**

Ivor-Lewis esophagectomy (ILE) is the standard surgical care for esophageal cancer patients but postoperative morbidity impairs quality of life and reduces long-term oncological outcome. Two-stage ILE separating the abdominal and thoracic phase into two distinct surgical procedures has proven to enhance microcirculation of the gastric conduit and therefore most likely reduces complications. However, two-stage ILE has not been evaluated systematically in selected groups of patients scheduled for this procedure. This investigation aims to demonstrate the feasibility of two-stage ILE in high-risk patients.

**Patients and methods:**

In this retrospective analysis of data obtained from a prospective database, a consecutive series of 275 hybrid ILE (hILE) were included. Patients were divided into two groups based on one- or two-stage hILE. Postoperative complications were assessed according to ECCG (Esophageal Complication Consensus Group) criteria and compared using the Clavien–Dindo score. Indication for two-stage esophagectomy was classified as pre- or intraoperative decision.

**Results:**

34 out of 275 patients (12.7%) underwent two-stage hILE. Patients of the two-stage group were significantly older. In 21 of 34 patients (61.8%) the decision for a two-stage procedure was made prior to esophagectomy, in 13 (38.2%) patients intraoperatively after completion of the laparoscopic gastric mobilization. The most frequent preoperative reason to select the two-stage procedure was a stenosis of the coeliac trunc and superior mesenteric artery (*n* = 10). The predominant cause for an intraoperative change of strategy was a laparoscopically diagnosed hepatic fibrosis/cirrhosis (*n* = 5).Overall morbidity and major’ complications (CD > IIIa) were comparable for both groups (11.7% in both groups). The overall anastomotic leak rate was 12.4% and was non-significant lower for the two-stage procedure.

**Conclusion:**

Two-stage hILE is a feasible concept to individualize the surgical treatment of patients with well-defined clinical risk factors for postoperative morbidity. It can also be applied after completion of the abdominal phase of IL esophagectomy without compromising the patient safety.

Despite recent improvements of perioperative management including advances of surgical techniques and postoperative care, esophagectomy remains a complex surgical procedure which is associated with a significant rate of morbidity and mortality [1]. However, centralization of services has seen mortality from esophagectomy decreasing to less than 5% in high volume centers but major morbidity remains high even in this setting [2]. There is mounting evidence that a complicated postoperative course impairs not only health-related quality of life but also has a negative impact on long-term oncological outcome [2, 3]. Therefore, one of the main goals remains to identify preoperative variables which accurately predict postoperative outcome. In a recent registry analysis, a laparoscopic approach and operations performed in high volume centers were identified as protective factors, whereas age, high comorbidities and squamous cell carcinoma were independent predictors of mortality [4].

In an attempt to reduce the surgical trauma, minimally invasive esophagectomy (MIE) has been introduced and developed over the last decade. However, despite its increasing application the scientific evidence supporting superiority of MIE compared to open esophagectomy is limited with only three randomized controlled trials [5–8] and some results derived from large national register analyses [9–12]. Beside the reduction of the intraoperative trauma by MIE, another theoretical approach to reduce the procedure-related morbidity is to split up the abdominal and thoracic phase of esophagectomy into two distinct surgical procedures with an interval of several days in-between. This concept also known as ischemic conditioning of the gastric conduit separates the gastric mobilization with partial devascularization from the gastric tube formation and pull-up during the thoracic part of esophagectomy [13, 14]. Several animal and clinical studies could proof the feasibility of this concept and demonstrated improved gastric microcirculation at the time of delayed reconstruction [15–17]. However, in larger retrospective series, the concept failed to demonstrate a reduction of postoperative morbidity since it was mainly applied for an unselected group of patients scheduled for esophagectomy [18, 19].

Therefore, this study was conducted to investigate whether two-stage IL esophagectomy is a feasible strategy in a selected group of patients with an increased risk for postoperative complications and to compare postoperative outcome with benchmark data.

## Patients and methods

### Study design

From 01.05.2016 to 30.04.2018, a total of 348 patients underwent an IL esophagectomy at the Department of General, Visceral and Cancer Surgery, University of Cologne. Patients undergoing open gastric mobilization (*n* = 44) and total minimally invasive esophagectomy (*n* = 23) were excluded. Furthermore, patients with a benign or malignant tumor other than squamous cell or adenocarcinoma of the esophagus or gastroesophageal junction were excluded (*n* = 6). The final study cohort consisted of 275 patients undergoing a hybrid IL esophagectomy. Patients were stratified in two groups according to application of one- or two-stage IL esophagectomy.

The study was designed as a feasibility trial based on a retrospective analysis with data obtained from a prospective database. The local Institutional Review Board approved the data collection. Patient consent for data analysis could be waived because individual patients were not identified.

### Surgery

Two-stage hybrid IL esophagectomy consisted of two separated surgical procedures. The first operation included the complete laparoscopic gastric mobilization with abdominal lymphadenectomy. The fatty tissue along the lesser curvature remained untouched and gastric tube formation was not initiated during the abdominal phase. Lymph node dissection was done after incision of the lesser omentum along the common hepatic artery and splenic artery, followed by dissection of the left gastric artery with nodal clearance of the retroperitoneal space up to the lower mediastinum (modified D2-lymphadenectomy). The dissected tissue remained attached to the lesser curvature. The gastroesophageal junction was completely mobilized at the level of the diaphragmatic hiatus. Devascularisation also included dissection of the short gastric arteries along the gastric fundus. Finally, the greater curvature was completely mobilized with visualization and preservation of the right gastroepiploic vessels. After an interval of 3–5 days right-sided open transthoracic esophagectomy was performed with dissection of the mediastinal lymph nodes (2-field lymphadenectomy). For patients with squamous cell carcinoma, lymphadenectomy was extended to compartments on left side of the trachea (extended 2-field lymphadenectomy). After pull-up of the stomach, the gastric tube was fashioned with a width of 4 cm using several magazines of a longitudinal stapler (45 and 60 mm longitudinal Endo-GIA (Medtronic®) stapling devices). After placing a purse string suture, esophagogastrostomy was done as end-to-site anastomosis with a 25 or 28 mm circular stapler (EEA, Medtronic®) above the level of the dissected transverse azygos vein. The anastomosis was located at the anterior wall of the gastric corpus closely to the greater curvature. After placing several tension-release sutures, the circular anastomosis was covered by an omental flap.

One-stage hybrid IL esophagectomy comprised the same surgical procedure except for the step that during the abdominal phase the fatty tissue on the lesser curvature was dissected at the level of incisura angularis and gastric tube formation was initiated laparoscopically (45 and 60 mm longitudinal Endo-GIA (Medtronic®) stapling devices).

All patients were extubated in the operating theater and transferred to the ICU for further recovery.

### Data collection and statistics

Prospectively collected data included basic demographics, American Society of Anesthesiologists (ASA) and World Health Organization (WHO)/Eastern Cooperative Oncology Group (ECOG) scores, body mass index (BMI) as well as comorbidities, tumor-specific parameters (histology, neoadjuvant therapy, pTNM stage, pathological regression) and technical details of the operation. In addition, all patients were preoperatively screened for atherosclerotic stenosis of the celiac trunk (TC) and the superior mesenteric artery (SMA) using computed tomography (CT) scans.

The indications for performing a two-stage esophagectomy were classified as preoperative (comorbidities, stenosis of the TC/SMA, systemic atherosclerosis) and intraoperative (respiratory or cardiac complications, hepatic fibrosis, gastric perfusion). In addition, the time interval between the two operations was recorded.

Postoperative complications were assessed according to ECCG (Esophageal Complication Consensus Group) definitions [20] and were classified according to the Clavien–Dindo (CD) Score [21]. Postoperative complications with a CD score ≤ IIIa were classified as, minor’, complications with a CD score > IIIa as, major’. A CD score of V was defined as in-hospital mortality. Recorded surgical complications were anastomotic leakage, conduit necrosis, chylous leakage, delayed gastric conduit emptying and the need for endoscopic interventions or re-operation. Furthermore, readmission to ICU and hospital stay (in days) were documented.

Data analysis was done retrospectively using IBM SPSS statistics software (version 25.0, SPSS Inc. Chicago). Statistical analysis was primarily based on descriptive means. Categorical data were summarized as frequencies and percentages. Continuous variables were analyzed using medians with corresponding interquartile range (IQR) and means with corresponding standard deviation. Statistical differences between the one- and two-stage esophagectomy groups were assessed using Pearson’s chi-square test, Fisher’s exact test or the Independent sample *t* test. *P* values less than 0.05 were considered statistically significant.

## Results

### Patient characteristics

Two hundred and forty one patients (87.3%) underwent a one-stage IL esophagectomy and 34 patients (12.7%) a two-stage IL esophagectomy. The demographic and baseline characteristics of the two groups were similar (Table 1) except for a significantly higher age in the two-stage group (mean of 69.0 vs. 61.6 years, *p* < 0.001). The BMI, ASA and ECOG scores of the two groups were also comparable. There were no significant differences regarding the distribution of the histology. If administered, the predominant neoadjuvant therapy was radiochemotherapy (CROSS protocol) in both groups (two-stage IL esophagectomy 72.7% vs. one-stage IL esophagectomy 77.1%, *p* = 0.409). Patients of the two-stage group demonstrated a lower rate of preoperative chemo- or chemoradiotherapy (14.2%, vs. 35.3% *p* = 0.019) and had a significant more advanced tumor stage at final pathological assessment (UICC ≥ III 50.0% vs.32.8%, *p* = 0.049).

**Table 1:** Demographic and baseline characteristics of 275 patients with hybrid IL esophagectomy

Characteristics	All patients		One-stage		Two-stage		*P*
	(*n* = 275)		(*n* = 241)		(*n* = 34)		
Age (years, mean ± SD)	62.6	± 10.5	61.6	± 10.3	69.0	± 9.9	< 0.001
BMI (kg/m^2^, mean ± SD)	26.9	± 5.0	27.0	± 4.9	26.3	± 5.5	0.467
Sex							0.378
Male (*n*, %)	228	82.9	198	82.2	30	88.2	
Female (*n*, %)	47	17.1	43	17.8	4	11.8	
ASA							0.062
1 (*n*, %)	17	6.2	13	5.4	4	11.8	
2 (*n*, %)	128	46.7	119	49.5	9	26.5	
3 (*n*, %)	125	45.6	105	43.8	20	58.8	
4 (*n*, %)	4	1.5	3	1.3	1	2.9	
ECOG							0.492
0 (*n*, %)	116	46.6	103	47.7	13	39.4	
1 (*n*, %)	96	38.6	83	38.4	13	39.4	
2 (*n*, %)	27	10.8	21	9.7	6	18.2	
3 (*n*, %)	10	4.0	9	4.2	1	3.0	
Histology							0.674
AC (*n*, %)	219	79.6	191	79.3	28	82.4	
SCC (*n*, %)	56	20.4	50	20.7	6	17.6	
Neoadjuvant therapy							
No (*n*, %)	46	16.8	34	14.2	12	35.3	0.019
Yes (*n*, %)	227	83.2	205	85.8	22	64.7	
RCTx (*n*, %)	174	76.7	158	77.1	6	27.3	
CTx (*n*, %)	53	23.3	47	22.9	16	72.7	
Pathological T stage							0.109
pT0 (*n*, %)	47	17.1	44	18.3	3	8.8	
pT1 (*n*, %)	54	19.6	46	19.0	8	18.6	
pT2 (*n*, %)	47	17.1	44	18.3	3	8.8	
pT3 (*n*, %)	122	44.4	104	43.2	18	52.9	
pT4 (*n*, %)	5	1.9	3	1.2	2	5.9	
Pathological N stage							0.066
pN0 (*n*, %)	146	53.1	132	54.8	14	41.2	
pN1 (*n*, %)	59	21.5	53	22.0	6	17.6	
pN2 (*n*, %)	35	12.7	30	12.4	5	14.7	
pN3 (*n*, %)	35	12.7	26	10.8	9	26.5	
UICC							0.049
0–II (*n*, %)	179	65.1	162	67.2	17	50.0	
III–IV (*n*, %)	96	34.9	79	32.8	17	50.0	
Resection margin							0.374
R0 (*n*, %)	263	95.6	232	96.3	31	91.2	
R1 (*n*, %)	12	4.4	9	3.7	3	8.8	

*ILE* Ivor-Lewis esophagectomy, *BMI* Body Mass-Index, *SD* standard deviation, *ASA* American society of anesthesiologists, *AC* adenocarcinoma, *SCC* squamous cell carcinoma, *RTCx* radiochemotherapy, *CTx* chemotherapy

### Indication for two-stage esophagectomy

In 21 of 34 patients (61.8%), the decision for a two-stage procedure was made prior to esophagectomy (Table 2). The most frequent indication was a stenosis of the coeliac trunk (TC) (10 of 21 patients, 47.6%). In 5 patients, CT scan revealed an isolated TC stenosis; in 5 patients, TC stenosis was diagnosed in combination with a stenosis of the superior mesenteric artery (SMA). In one patient, two-stage IL esophagectomy was performed due to a general atherosclerosis. In 10 patients (47.6%) scheduled for the two-stage procedure, decision was made due to a borderline functional reserve related to multiple comorbidities, predominantly respiratory and cardiac dysfunction.

**Table 2:** Indication for two-stage hybrid IL esophagectomy in 34 patients

	No	%
Intraoperative	13	38.2
Suspected metastases	1	2.9
Cardiac	1	2.9
Pulmonary	3	8.8
Gastric perfusion	3	.8.8
Hepatic	5	14.7
Preoperative	21	61.8
General atherosclerosis	1	2.9
Multiple comorbidities	10	9.4
SMA/TC stenosis	10	29.4
TC stenosis only	5	14.7
Both	5	14.7

*SMA* superior mesenteric artery, *TC* coeliac trunc

In 13 patients (38.2%), a one-stage IL esophagectomy was intended but changed to a two-stage procedure during the laparoscopic phase of IL esophagectomy (Table 2). The most frequent cause for this change of strategy was a macroscopic evidence of hepatic fibrosis during laparoscopy (5 of 13 patients, 14.7%). Other indications were intraoperative cardiopulmonary complications (11.7%), an impaired perfusion at the anastomotic site of the gastric fundus after gastric devascularization (8.8%) and suspected metastasis (2.9%) which could not be clearly confirmed by frozen section.

### Postoperative outcome

The postoperative outcome of patients following one- and two-stage esophagectomy is summarized in Table 3. The median interval between laparoscopy and thoracotomy in the two-stage group was four days (IQR 4–5 days). One hundred patients of the one-stage and 12 patients of the two-stage groups had an uneventful postoperative course (CD 0, 41.7% vs. 35.3%).Minor’ complications (CD ≤ IIIa) occurred in 112 patients (46.7%) of the one-stage and in 18 patients of the two-stage groups (52.9%). There was also no difference with respect to, major’ complications (CD > IIIa) (28/241 patients (11.6%)) in the one-stage group vs 4/34 patients (11.8%) in the two-stage group.

**Table 3:** Postoperative outcome in 275 patients with hybrid IL esophagectomy

Characteristics	All patients		One-stage		Two-stage		*p*
	(*n* = 275)		(*n* = 241)		(*n* = 34)		
Dindo–Clavien							0.118
0 (*n*, %)	112	40.9	100	41.7	12	35.3	
I (*n*, %)	13	4.6	10	4.1	3	8.8	
II (*n*, %)	21	7.7	17	7.1	4	11.8	
IIIa (*n*, %)	96	35.0	85	35.4	11	32.4	
IIIb (*n*, %)	11	4.0	11	4.6	0	0.0	
IV (*n*, %)	20	7.3	17	7.1	3	8.8	
V (*n*, %)	1	0.4	0	0.0	1	2.9	
Anastomotic leak							0.503
No (*n*, %)	241	87.6	210	87.1	31	91.2	
Yes (*n*, %)	34	12.4	31	12.9	3	8.8	
Type I (*n*, %)	1	2.9	1	3.2	0	0	
Type II (*n*, %)	24	70.6	22	71.0	2	66.7	
Type III (*n*, %)	9	26.5	8	25.8	1	33.3	
Chyle leak							0.058
No (*n*, %)	270	98.2	238	98.8	32	94.1	
Yes (*n*, %)	5	1.8	3	1.2	2	5.9	
Readmission ICU							0.403
No (*n*, %)	236	87.1	209	87.8	28	82.4	
Yes (*n*, %)	35	12.9	29	12.2	6	17.6	
Interval (d, median, IQR)					4	4–5	
ICU stay total (d, median, IQR)	2	2–5	2	2–5	3.5	2–6	0.252
Hospital stay (d, median, IQR)	14	13–20	14	13–20	16.5	12–23	0.130

*ILE* Ivor-Lewis esophagectomy, *ICU* intensive care unit, *OP* operation, *IQR* interquartile range

The overall leakage rate in this patient cohort was 12.4% (34 of 275 patients). The majority of leakages (24 of 34 patients) were treated successfully by endoscopy using an Eso-sponge or a metal stent (ECCG type II leakage) and 9 of 34 patients underwent re-operation (ECCG type III leakage).The rate of anastomotic leaks was lower in the two-stage esophagectomy group without reaching statistical significance (8.8% vs. 12.9%, *p* = 0.503). The distribution of ECCG type II and type III leakages were comparable for one-stage and two-stage esophagectomy (Table 3).

*The overall chylous leakage rate was 1.8% (5 of 275 patients) without any significant differences in both cohorts (p* = *0.058), though it occurred more frequently in the two-stage group (2/32 patients (5.9%)) than in the one-stage group (3/238 patients (1.2%)).*

There were neither any major intraoperative and postoperative bleedings nor major conduit ischemia observed as well as the readmission rate to ICU (12.2% in the one-stage vs. 17.6% in the two-stage group, *p* = 0.403), and the median overall hospital stay (14 days in the one-stage vs. 16.5 days in the two-stage, *p* = 0.130).

## Discussion

Transthoracic esophagectomy as standard treatment for esophageal cancer remains a complex surgical procedure associated with a high rate of postoperative complications of up to 60% but with a low mortality rate in specialized centers [1, 22]. Therefore, the predominant goal of perioperative management is to reduce postoperative morbidity aiming to improve patient quality of life and long-term oncologic outcome. For the perioperative period, prehabilitation and fast track programs are under investigation to accelerate recovery, thereby reducing postoperative complications [23, 24]. For the same reason, minimally invasive esophagectomy with manifold technical variations has been introduced but despite its widespread application there is only little evidence to demonstrate the superiority of minimally invasive approaches [5–12].

A two-stage approach as described in this analysis with separation of the abdominal and thoracic phase is an additional strategy to reduce morbidity of IL esophagectomy. The general surgical strategy is almost identical between one- and two-stage IL esophagectomy. The only difference relates to formation of the gastric tube which is completely performed during the second (thoracic) part of the two-stage approach, whereas it usually initiated at the end of the laparoscopic gastric mobilization. The postponement of the reconstructive phase appears necessary since the diversion of the lesser curvature with a longitudinal stapler inevitably results in a partial necrosis of the stapled and devascularized short lesser curvature segment with subsequent risk of gastric perforation. During laparoscopy, dissection of the fatty tissue on the lesser curvature with separation of the vascular supply between right and left gastric artery is an optional but not necessary step of devascularisation because its contribution to overall gastric perfusion is minor.

In this large consecutive series of esophageal cancer patients treated in a high volume center, one-stage hybrid IL esophagectomy presents the standard of surgical care. However, one out of ten patients underwent a two-stage procedure. The novelty of this concept comprises the expansion from the preoperative to the intraoperative indication depending on the intraoperative morphological findings as well as the patient condition under general anaesthesia. Summarizing the indications, the pre- and intraoperative decision making for two-stage esophagectomy can be classified into two main groups.

The first group consists of those patients having an increased risk of inadequate perfusion to the gastric conduit due to changes of micro- or macrocirculation. There is mounting evidence that atherosclerotic changes of the celiac trunk and/or superior mesenteric artery are associated with an increased risk of anastomotic leakage. In a consecutive series of 145 patients scheduled for Ivor-Lewis esophagectomy the overall incidence of coeliac trunc stenosis defined as any atherosclerotic narrowing on CT scan was 40% [25]. The incidence of anastomotic leak in patients with stenosis was 19.4% compared to 2.3% in patients without stenosis and multivariable analysis identified stenosis as independent risk factor of leakage. The results were confirmed by a second observational study using another well-defined calcification score as indicator for anastomotic complications [26, 27]. Although it is not known which score should be used for assessment [28], the authors of both studies concluded that routine assessment of the staging CT for possible atherosclerotic changes of the coeliac trunk and gastric arteries is recommended. Two-stage esophagectomy as performed in this series represents a possible treatment option for patients with this well-defined risk factor. This strategy is also supported by anastomotic leakage rate which is comparatively low for patients treated with a two-stage approach. In addition, in some patients, the gastric fundus appears not to be well perfused at the end of the laparoscopic gastric mobilization. This intraoperative diagnosis might be confirmed using novel techniques like intraoperative Indocyanine green (ICG) application to visualize the gastric perfusion. ICG-based assessment of gastric perfusion is still under development and thresholds of inadequate microcirculation safely predicting anastomotic failure are not known at the present [29]. Patients without preoperative evidence of any atherosclerotic changes presenting with intraoperative suspect of impaired perfusion were also included into the two-stage approach and seem to experience a similar benefit.

The second group (29.4%) of indications for two-stage esophagectomy comprised patients with multiple concomitant comorbidities as expressed by advanced age. All of these scores proved to have an inversely proportional correlation with early postoperative outcome measured by the overall morbidity and mortality rate or the Clavien–Dindo score [4]. Despite these well-established scores, a severely impaired function of single organs in particular the respiratory, cardiac and hepatic system are known to be associated with a more complicated postoperative course. However, for many of these patients with single organ dysfunction, it is still difficult to predict the incident of intra- or postoperative organ deterioration. Two-stage esophagectomy offers the possibility to split up the surgical trauma and therefore potentially reduce the stress to the impaired organ systems. Furthermore, it is also possible to withdraw from the transthoracic procedure in case of an acute organ failure even after completion of the laparoscopic gastric mobilization. This enables surgeons to further evaluate patients with a borderline functional operability. Moreover, results for this high-risk patient cohort as defined by the Clavien–Dindo score are comparable to those of the general cohort receiving a one-stage Ivor-Lewis esophagectomy or recently published benchmarking data [1, 22].

## Conclusion

Two-stage esophagectomy is a feasible concept to individualize the surgical treatment of patients with a high risk for postoperative complications. Based on these results, two-stage esophagectomy does not compromise patient safety. The two-stage procedure seems to be particularly beneficial in patients with risk factors of an impaired vascular gastric perfusion or patients with borderline functional operability due to single or multiple organ dysfunctions. The decision to follow a two-stage strategy can be made before esophagectomy or after completion of the laparoscopic gastric mobilization and therefore increases the flexibility and individualization of the surgical treatment. This study provides evidence to further evaluate the two-stage approach in a prospective study.
